# Is there an association between perceived social support and cardiovascular health behaviours in people with severe mental illnesses?

**DOI:** 10.1007/s00127-020-01879-9

**Published:** 2020-05-18

**Authors:** Alexandra Burton, Kate Walters, Louise Marston, David Osborn

**Affiliations:** 1 Division of Psychiatry, University College London, Maple House, 6th floor, 149 Tottenham Court Road, London, W1T 7NF UK; 2 Department of Primary Care and Population Health, University College London, UCL Medical School (Royal Free Campus), Upper Third Floor, Rowland Hill Street, London, NW3 2PF UK

**Keywords:** Severe mental illness, Cardiovascular disease, Social support, Primary care

## Abstract

**Purpose:**

People with severe mental illnesses (SMI) have an increased risk of cardiovascular disease (CVD). Research in the general population suggests that social support may protect against increased CVD morbidity and mortality; however, this may not apply to those with SMI. We aimed to explore the association between perceived social support and attendance at primary care nurse CVD risk reduction clinic appointments and CVD risk-reducing behaviours in an SMI population with elevated CVD risk factors.

**Methods:**

We used longitudinal and cross-sectional data from a randomised controlled trial on 326 adults with SMI recruited via 76 general practices in England. Multilevel regression analysis estimated the effect of perceived social support on attendance at CVD risk reduction clinic appointments over 6 months, and adherence to CVD medication, physical activity, diet, smoking and alcohol use at baseline, adjusted by age, sex, ethnicity, deprivation, psychiatric diagnosis and employment.

**Results:**

Perceived social support predicted greater appointment attendance in unadjusted (IRR = 1.005; 1.000–1.010; *p* = 0.05) but not adjusted analysis (IRR = 1.003; 0.998–1.009; *p* = 0.25). Perceived social support was associated with greater adherence to medication; for each 1% increase in social support, there was a 4.2% increase in medication adherence (OR = 1.042; 1.015–1.070; *p* = 0.002). No association was found between greater perceived social support and greater physical activity, lower sedentary behaviour, healthier diet, lower alcohol use or being a non-smoker.

**Conclusions:**

Social support may be an important facilitator for CVD medication adherence and is potentially important for primary care appointment attendance; however, alternative strategies might be needed to help people with SMI engage in physical activity, healthier diets and to reduce their smoking and alcohol use.

## Introduction

People with severe mental illnesses (SMI) such as schizophrenia and bipolar disorder are at an increased risk of cardiovascular disease (CVD) and die up to 20 years earlier than the general population [1]. The mortality gap between people with SMI and the general population is widening [2]. Factors which may be responsible for this health inequality include increased smoking rates, poor diet and sedentary lifestyles [3, 4], a high rate of diabetes [5], side effects of antipsychotic medications [6] and sub-optimal management by health professionals of CVD risk in this population [7, 8]. The importance of monitoring and improving the physical health of people with SMI is endorsed by national and international guidelines [9, 10]; however, a UK survey of people with schizophrenia found that only 33% of respondents had attended a full CVD screening appointment in the last year [11].

One factor that may influence cardiovascular health outcomes is the availability of social support and the degree to which an individual perceives that they are integrated within a social network. There is a wealth of research in the general population indicating that people with low levels of social support have worse health outcomes than those who have positive social relationships including an increased risk of mortality in people with CVD [12, 13], diabetes [14] and hypertension [15]. Theories suggest that social support either has a direct impact on cardiovascular health through its influence on social norms and behaviours such as cigarette smoking, alcohol use, dietary intake and physical activity, or an indirect or “buffering” effect whereby the presence of social support reduces stress, which in turn strengthens cognitive, emotional and physiological responses to illness [16, 17]. Measures of social support which assess the quality of relationships and the perceived availability of support if and when it is needed better predict health outcomes than measures of received support (whereby the level of support is assessed in response to current scenarios or life events), or binary measures such as living alone or marital status [12].

Research with SMI populations has found associations between greater social support and improved psychiatric outcomes including fewer psychiatric relapses [18, 19] and greater adherence to psychiatric medications [20, 21]. Qualitative studies have identified a lack of social support as a barrier for physical activity [22–24] and quitting smoking [25, 26] in people with SMI; however, only one observational study identified an association between greater social support and physical activity [27]. A small number of studies found no association between social support and physical activity [28, 29], diet [28], smoking [30, 31] or alcohol use [32, 33] in people with SMI. Limitations of these studies included a lack of validated measures of social support, use of non-global measures to assess social support and small sample sizes. Only one study took place within a UK health setting [32]. To our knowledge, no studies currently exist on the relationship between perceived social support and attendance at CVD health-related appointments or adherence to CVD risk-reducing medications in SMI populations.

Our primary aim was to investigate whether higher perceived social support was associated with attendance at a greater number of primary care practice nurse/health care assistant (HCA) CVD health promotion appointments over 6 months in people with SMI and raised CVD risk factors. The secondary aims were to test whether there were cross-sectional associations between higher perceived social support and greater adherence to CVD medications, increased physical activity, lower sedentary behaviour, healthier diet, being a non-smoker and lower alcohol use.

## Method

### Procedure

We conducted a secondary analysis of data collected as part of the PRIMROSE RCT which tested the effectiveness of a CVD risk-reducing intervention for people with SMI in primary care [33, 34]. The intervention consisted of appointments with a practice nurse or HCA over 6 months and aimed to reduce CVD risk factors including raised cholesterol, smoking, unhealthy diet, alcohol use and low levels of physical activity in people with SMI who had two or more risk factors for CVD. The comparison group received treatment as usual. We tested for an association between perceived social support at baseline and attendance at PRIMROSE primary care intervention appointments at 6-month follow up. Cross-sectional analyses were conducted to assess whether there were associations between perceived social support and self-reported adherence to CVD risk-reducing medications, physical activity, diet, alcohol use and smoking at baseline. Data collection was approved by the City Road and Hampstead Research Ethics Committee (Reference No. 12/LO/1934, 10 January 2013) as part of the PRIMROSE trial NRES committee application.

### Participants

The sample consisted of 326 patients aged 30–75 years old who were included on the SMI register at one of 76 GP practices recruited to the PRIMROSE trial [34, 35]. All recruited participants had a diagnosis of schizophrenia, persistent delusional disorder, schizoaffective disorder, bipolar affective disorder, psychosis, psychotic depression or other psychotic disorder recorded in their GP medical record. The trial inclusion criteria were a raised total cholesterol above 5 mmol/l or raised total cholesterol/HDL cholesterol ratio above four; and one or more other CVD risk factors including: BMI > 30 kg/m^2^, current smoker, blood pressure > 140 mmHg systolic and/ or > 90 mmHg diastolic on two or more consecutive occasions, HbA_1c_ of 42–47 mmol/mol (6.0–6.4%), diagnosis of diabetes or hypertension.

Participants were excluded from the study if they were currently in an inpatient unit or accessing a crisis service, had a diagnosis of an organic mental health problem, personality disorder and/or severe cognitive impairment, life expectancy of less than 6 months, were pregnant or had pre-existing CVD.

### Measures

Data were collected by research nurses working in clinical research networks (CRNs) in England from three sources (1) the participant’s medical record (2) researcher-administered questionnaires and (3) self-complete patient questionnaires. Attendance at the PRIMROSE intervention appointments was collated by practice nurses or HCAs based in recruited GP practices.

### Demographics and descriptive data

We recorded participants’ sex, ethnicity, date of birth, marital status, social network size, living arrangements, employment status, Townsend deprivation quintile and whether they had a support worker. Primary psychiatric diagnosis was taken from the participant’s GP medical record. All other demographic data were collected directly from the participants using researcher-administered questionnaires.

### Perceived social support

Perceived social support was measured using the Medical Outcomes Study: Social Support Survey—MOS-SSS [36]. The MOS-SSS is a validated and widely used self-report measure of the perceived availability of functional social support (i.e., whether social support would be available if and when it was needed) [37]. The MOS-SSS has been used to assess perceived social support in populations with schizophrenia [38] and consists of 19 items each assessed using a five point Likert scale to determine whether participants feel that they are supported one “none of the time” to five “all of the time”. Questions assess the perceived availability of emotional and informational support, practical support, affection and positive interactions. An overall functional support index score is generated by calculating the average score across the 19 items in the scale. The range for the overall score is 1–5 which was then converted using a formula developed by the paper authors, so that the lowest possible score was 0 and the highest possible score was 100.

The measure is reliable (Cronbach alpha coefficient = 0.97) and stable over time [36].

### Attendance at PRIMROSE CVD risk reduction intervention appointments

Attendance at appointments was only assessed in the PRIMROSE intervention arm (*n* = 155 people). Practice nurses/HCAs delivering the PRIMROSE intervention were asked to complete an appointment attendance spreadsheet for each participating patient at their GP practice and indicate whether or not an appointment was scheduled and subsequently attended, or not attended. Practice nurses/HCAs were asked to deliver a minimum of eight and a maximum of 12 appointments to each patient over a 6-month period. Practice nurses/HCAs were advised to see participants every 1–2 weeks for their first five intervention appointments, reducing to every 2–4 weeks for the remaining appointments.

### CVD medication adherence

Adherence to medication was measured using the validated Morisky Medication Adherence Scale (MMAS-8)^1^ [39, 40]. The scale has been widely used in research with hypertensive and diabetic populations [41, 42]. It is a patient self-complete questionnaire that can be used to ask participants about specific medication use and was administered in this study to ask participants specifically about their adherence to CVD risk-reducing medications such as statins, antihypertensives, metformin, stop-smoking medication and/or diabetic medications. The scale contains eight questions; the first seven of which are yes/no responses and the final item is a five point Likert response. A total score of 0–8 is possible with higher scores indicating greater medication adherence.

### Physical activity

Physical activity was assessed using the International Physical Activity Questionnaire (IPAQ) short form [43], a widely used self-complete questionnaire which has been validated in people with schizophrenia [44, 45] The questionnaire has been shown to demonstrate good test–retest reliability, and reasonable concurrent and criterion validity [43]. It is structured so that it gives separate scores in three domains: walking, moderate intensity activity and vigorous intensity activity. The final question asks participants to indicate how much time they spend sitting on a typical day during the last 7 days.

### Diet

Diet was assessed using the Dietary Instrument for Nutrition Education (DINE), a validated food frequency questionnaire which is administered as a structured interview [46, 47]. The DINE was developed and validated in a sample of 206 primary care attenders in the UK and has been used to assess dietary behaviour in previous research studies with people with SMI [48]. Questions were asked on the frequency that 19 specific foods were eaten by the participant, organised into total fat, fibre and unsaturated fat food groups. Separate overall scores were then calculated for each food group with a higher score on each respective food group (fat, fibre and unsaturated fat) indicating a greater intake of that specific food group and categorised as either low, medium or high consumption. The questionnaire does not allow for an overall dietary score to be calculated.

### Alcohol use

Alcohol use was measured using the Alcohol Use Disorders Identification Test (AUDIT) [49]. It has been validated and found to be reliable in studies involving people with SMI [50, 51] and is widely used in UK clinical practice to assess whether a patient is at risk of alcohol misuse problems. It consists of 10 questions with the first three questions measuring frequency and quantity of alcohol use. If a score of five or more is obtained, participants are then asked the remaining seven questions which explore the perceived consequences of the participant’s alcohol use. A score of 0–40 is possible with higher scores indicating increasing risk of alcohol dependency.

### Smoking

Participants were asked their current smoking status: (1) non-smoker, (2) ex-smoker, (3) light smoker (9 or less cigarettes a day), (4) moderate smoker (between 10 and 19 cigarettes a day) or (5) heavy smoker (20 or more cigarettes a day). Answers were converted in to a binary outcome and participants were categorised as either (1) current smokers or (2) non-current smokers.

### Statistical methods and data analysis

We used Stata Version 14 [52] to carry out the statistical analyses. Summary statistics for all variables were produced. For continuous variables, the mean and standard deviation or median and interquartile range were computed as appropriate. Summary statistics for categorical variables were reported as frequency and percentage within each category.

Continuous independent and dependent variables were explored for normality using histograms and, following modelling, residual plots were generated. Unadjusted analyses were then performed between the independent variable of perceived social support as measured by the MOS-SSS questionnaire and each pre-specified dependent variable using random effects logistic regression. Negative binomial regression was used for count outcomes that were over dispersed. Adjusted analyses were then performed, firstly entering sex and age into the model and then sex, age, ethnicity, psychiatric diagnosis, deprivation and employment. The analyses accounted for clustering as a random effect at the level of the GP practice.

The analysis on the primary outcome (attendance at PRIMROSE intervention appointments) was conducted on the intervention group sample only as the control group did not receive the intervention. All other analyses were performed on the combined baseline data from both the intervention and usual care groups treated as one sample.

### Covariates

Based on previous research on predictors of social support and CVD risk-reducing behaviours in people with SMI, we included the following covariates in the adjusted models: sex, age, ethnicity, psychiatric diagnosis, deprivation and employment status. Previous research suggested that sex [53, 54], age [53, 55], ethnicity [54, 55] and employment [55] were associated with social support in SMI. Studies have also found that sex [27], age [29, 56] and psychiatric diagnosis [27] were predictors of physical activity in people with SMI, and age [30, 57], and ethnicity [58] were predictors of smoking. No studies were found on predictors of attendance at health appointments, adherence to CVD risk-reducing medications, diet or alcohol use in SMI. Deprivation was included as a plausible factor that may have an impact on social support and CVD health behaviours (i.e., those from deprived areas may have less social support and participate in fewer CVD risk-reducing behaviours than those from less deprived backgrounds).

## Results

### Descriptive analysis

**Fig. 1:**
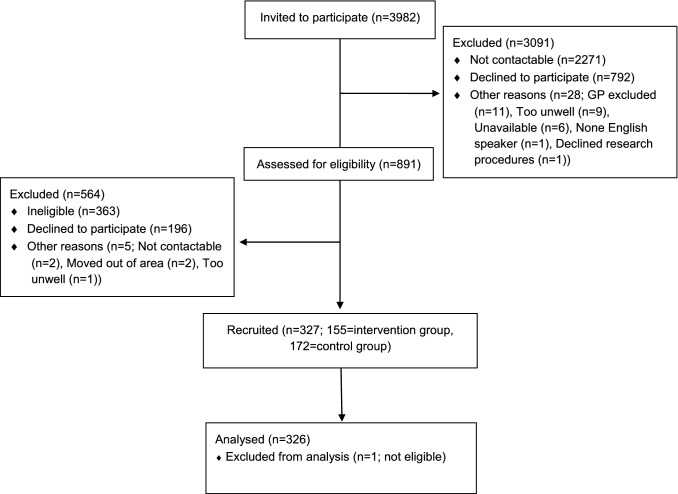
Participant recruitment flow diagram

327 participants were recruited to the study across 76 GP practices with a mean of 4.3 patients and a range of 1–10 patients recruited per GP practice. 155 participants were randomised to the intervention group and 172 to treatment as usual. One patient in the treatment as usual group was identified as not eligible for the study and was, therefore, removed from the analysis. See Fig. 1 Participant Recruitment Flow Diagram for further details on the number of participants who were approached, were eligible and recruited to the study.

The amount of missing data for the independent and dependent variables were explored and a low proportion of missing data was identified. The largest amount of missing data was for the IPAQ total MET minutes score which six out of 326 participants did not complete (1.8% missing data). Complete case analysis was, therefore, used.

### Study sample

Characteristics of the PRIMROSE intervention group sample on which the primary analysis was conducted and the overall PRIMROSE sample on which the secondary analyses were conducted are presented in Table 1.

**Table 1:** Characteristics of the intervention group and overall samples

Variable	Intervention group		Overall sample	
	*n*/N or mean/median	Percent or SD/IQR	*n*/N or mean	Percent or SD
Gender				
Male	67/155	43.2	154/326	47.2
Age	50.9	10.0	50.8	9.9
Ethnicity				
White	134/154	87.0	289/325	88.7
Black	11/154	7.1	16/325	4.9
Asian	5/154	3.2	10/325	3.1
Other	4/154	2.6	10/325	3.1
Townsend deprivation quintile				
1 = Least deprived	22/136	16.2	39/255	12.0
2	7/136	5.1	18/255	5.5
3	17/136	12.5	28/255	8.6
4	30/136	22.1	58/255	17.8
5—most deprived	60/136	44.1	112/255	34.4
Marital status				
Single	66/154	42.9	133/324	41.1
Married or cohabiting or civil partners	59/154	38.3	123/324	38.0
Separated or divorced or civil partners	25/154	16.2	59/324	18.2
Widowed	4/154	2.6	9/324	2.8
Living arrangements				
With others	83/155	53.5	187/326	57.4
Lives alone	72/155	46.5	139/326	42.6
Employment				
Unemployed	71/155	45.8	147/326	45.1
Primary diagnosis				
Schizophrenia/schizoaffective	54/155	34.8	105/326	32.2
Bipolar	71/155	45.8	159/326	48.8
Other psychoses	30/155	19.4	62/326	19
Social support				
MOS-SSS	52.4	25.2	56	25.1
Primary outcome—appointment attendance				
Number of intervention appointments attended^a^	5	1,9	N/A	N/A
Secondary outcomes				
MMAS-8 (CVD prevention medication)				
High and moderate medication adherence	N/A	N/A	103/145	71
Low medication adherence	N/A	N/A	42/145	29.0
IPAQ (physical activity)				
Low activity	N/A	N/A	140/320	43.8
Moderate and vigorous activity	N/A	N/A	180/320	56.3
Sitting total MET minutes (Median and IQR)	N/A	N/A	360	(240, 480)
DINE (Diet)				
Fat intake				
Low fat intake	N/A	N/A	155/326	47.6
Medium/high fat intake	N/A	N/A	171/326	52.4
Fibre intake				
Low fibre intake	N/A	N/A	156/326	47.9
Medium/high fibre intake	N/A	N/A	170/326	52.1
Unsaturated fat intake				
Low unsaturated fat intake	N/A	N/A	16/326	4.9
Medium/high unsaturated fat intake	N/A	N/A	310/326	95.1
AUDIT (Alcohol)				
Low risk drinkers	N/A	N/A	247/326	75.8
Moderate, high risk or possible dependence	N/A	N/A	79/326	24.2
Smoking status				
Non-smoker	N/A	N/A	166/325	51.1
Current smoker	N/A	N/A	159/325	48.9

^a^median and interquartile range (IQR) reported

### Sample receiving the PRIMROSE trial intervention

The mean age of participants was 50.9 years old (standard deviation = 9.9), with 67 (43.2%) men randomised to the intervention group. 134/155 (87%) participants were white, 11/154 (7.1%) were black, 5/154 (3.2%) were Asian and 4/154 (2.6%) indicated that they were of “other” ethnicity. 54/155 (34.8%) participants had a primary diagnosis of schizophrenia or schizoaffective disorder, 71/155 (45.8%) had a diagnosis of bipolar disorder and 30/155 (19.4%) had a diagnosis of other psychosis. The mean perceived social support score on the MOS-SSS was 52.39 (SD = 25.23) with a range of 2.63–100.

### Total PRIMROSE trial sample

The mean age of participants was 50.8 years old, with 155 (47.5%) men taking part in the study. 289/325 (88.7%) participants were white, 16/325 (4.9%) were black, 10/325 (3.1%) were Asian and 10/325 (3.1%) indicated that they were of “other” ethnicity. 105/326 (32.2%) participants had a primary diagnosis of schizophrenia or schizoaffective disorder, 159/326 (48.8%) had a diagnosis of bipolar disorder and 63/326 (19.0%) had a diagnosis of other psychosis. The mean perceived social support score on the MOS-SSS was 55.96 (SD = 25.08) with a range of 2.63–100.

### Perceived social support and appointment attendance

The number of appointments attended ranged from 0 to 14 with 123/155 (79.4%) patients attending one or more appointments and 32/155 (20.6%) patients attending none.

An unadjusted negative binomial regression analysis was conducted to assess the relationship between social support and attendance at primary care appointments. For a one-point increase in score on the MOS-SSS, the rate of appointments attended increased by 0.5% (incident rate ratio = 1.005, 95% CI 1.000–1.011, *p* = 0.05).

When age and sex were entered into the model, the association between social support and attendance at primary care appointments was attenuated and no longer significant (IRR = 1.005, 95% CI 0.999–1.010, *p* = 0.09). This remained non-significant when all demographic variables (sex, age, ethnicity, psychiatric diagnosis, deprivation and employment) were entered into the fully adjusted model (IRR = 1.003, 95% CI 0.998–1.009, *p* = 0.25).

### Perceived social support and secondary outcomes

The unadjusted random effects logistic regression analysis found that for a one-point increase in perceived social support, the odds of being in the moderate/high adherence to medication group compared to the low adherence group increased by 3.9% (OR = 1.039, 95% CI 1.018–1.060, *p* < 0.001). The association remained when adjusted for sex and age (OR = 1.041, 95% CI 1.019–1.063, *p* < 0.001) and when fully adjusted for sex, age, ethnicity, psychiatric diagnosis, deprivation and employment (OR = 1.042, 95% CI 1.015–1.070, *p* = 0.002).

There was no significant association between social support and physical activity in the unadjusted analysis or in the analysis adjusted for sex and age; however, when sex, age, ethnicity, psychiatric diagnosis, deprivation and employment were entered into the model, the association became significant (OR = 0.989, 95% CI 0.978–1.000; *p* = 0.05).

No significant associations were detected between perceived social support and any other secondary outcomes (sedentary behaviour, diet, alcohol use, or smoking). The results of the unadjusted and adjusted analyses can be found in Table 2.

**Table 2:** Unadjusted and adjusted analyses on the association between perceived social support and secondary outcomes

	Medication adherence (MMAS-8) *n* = 145	IPAQ physical activity *n* = 320	IPAQ time spent sitting *n* = 322	DINE score fat *n* = 326	DINE score fibre *n* = 326	DINE score unsaturated fat *n* = 326	AUDIT score *n* = 326	Current smoker *n* = 325
Unadjusted analysis^b^	**1.039, (1.018–1.060; p < 0.001)**	0.993, (0.984–1.002; *p* = 0.13)	1.003, (0.993–1.013; *p* = 0.56)	1.000, (0.990–1.009; *p* = 0.91)	1.003, (0.995–1.012; *p* = 0.47)	1.002, (0.980–1.025; *p* = 0.87)	1.004, (0.994–1.015; *p* = 0.43)	1.006, (0.997–1.014; *p* = 0.22)
Adjusted for sex and age	**1.041, (1.019–1.063; p < 0.001)**	0.993, (0.998–1.002; *p* = 0.14)	1.003, (0.993–1.013; *p* = 0.56)	0.999, (0.999–1.008; *p* = 0.83)	1.003, (0.994–1.012; *p* = 0.55)	Analysis not performed^d^	1.004, (0.993–1.015; *p* = 0.51)	1.005, (0.996–1.004; *p* = 0.30)
Fully adjusted analyses^^^	**1.042, (1.015–1.070; p = 0.002)**	**0.989, (0.978–1.000; p = 0.05)**	1.005, (0.993–1.017; *p* = 0.41)	0.996, (0.985–1.008; *p* = 0.54)	0.998, (0.987–1.009; *p* = 0.73)	Analysis not performed^d^	1.004, (0.991–1.017; *p* = 0.58)	1.002, (0.991–1.013; *p* = 0.68)

Bold values indicate signficant associations

^a^*MMAS-8* morisky medication adherence scale, *IPAQ* International Physical Activity Questionnaire, *DINE* dietary instrument for nutrition education, *MET* metabolic equivalent of task, *AUDIT* alcohol use disorders identification test

^b^ Data are change in each outcome variable (odds ratio) for a one-point increase in perceived social support as measured by the MOS-SSS (95% CI; p value)

^c^Fully adjusted models include sex, age, ethnicity, psychiatric diagnosis, deprivation and employment status

^d^Due to the small number of participants categorised in the low unsaturated fat intake group (16/326), it was not possible to conduct the adjusted analyses

## Discussion

We found an association between perceived social support and attendance at primary care intervention appointments showing that each 1% increase in perceived social support was associated with a 0.5% increase in appointment attendance (IRR = 1.005; *p* = 0.05, 95% CI 1.000–1.011). Thus for every 10-point increase on the MOS-SSS (equivalent to a 10% difference on the scale), the attendance appointment rate was expected to increase by 5%. When sex, age, ethnicity, diagnosis, deprivation and employment were entered into the model, each 1% increase in perceived social support was associated with a 0.3% increase in appointment attendance and was no longer significant (IRR = 1.003; *p* = 0.25, 95% CI 0.998–1.009).

We identified an association between perceived social support and adherence to CVD medication. The odds of being in the moderate/high medication adherence group compared to the low adherence group increased by 3.9% with each 1% increase in perceived social support (OR = 1.039; *p* < 0.001, 95% CI 1.018–1.060). This association remained significant when all demographic variables where entered into the model (OR = 1.042; *p* = 0.002, 95% CI 1.015–1.070). There was no association between social support and physical activity in the unadjusted analysis or the analysis adjusted for sex and age, however, in the fully adjusted analysis, the result became significant, with higher perceived social support associated with lower odds of being in the moderate/vigorous activity group compared to the low physical activity group (OR = 0.989, 95% CI 0.978–1.000; *p* = 0.05). This association should be treated with caution and would need confirming in further work. No association was found between perceived social support and sedentary behaviour, diet, alcohol use or smoking status.

The findings highlight the potential importance of involving supportive others in CVD medication adherence and in identifying those who have low social support to help prevent disengagement and non-adherence to treatments. Social support may be particularly beneficial for supporting health behaviours that do not require supportive others to make significant changes to their own behaviour. Support with physical activity, diet, alcohol use and smoking may require changes in behaviour by both the participant and the person supporting them, and participants may therefore receive less support from others for changing these behaviours. It may, therefore, be less likely that social support has an impact on these particular health behaviours compared to medication adherence and appointment attendance which do not require changes in behaviour by the supportive other beyond monitoring and encouragement. The association between social support and medication adherence could also be explained via provision of practical assistance for medication taking such as supervision, monitoring or prescription collection on behalf of the patient [59] as well as approval from others of medication taking, which has been found to be important for adherence to smoking cessation medication in people with SMI [60].

The lack of association between perceived social support and physical activity, diet, smoking and alcohol use mirrors findings from previous studies in people with SMI [27, 28, 30, 32, 61]. Research with the general population has, however, shown that higher perceived social support is related to increased participation in healthy lifestyle activities [62–64]. The discrepancy between SMI and non-SMI populations could be because perceived social support has less impact on physical health outcomes in people with SMI, or that alternative measures of social support are more important e.g., received support, the number of people in the social network or peer support/support from professionals. The mechanisms for this are, however, unclear and require further research.

### Strengths, limitations and directions for future research

This was the first-known study seeking to identify an association between perceived social support and adherence to CVD risk-reducing medication, attendance at CVD health-promoting intervention appointments and participation in CVD health behaviours in people with SMI and raised CVD risk factors in a UK primary care setting. We selected validated questionnaires used in previous studies with people with SMI and in primary care.

The inclusion and exclusion criteria were originally designed for an interventional study aimed at reducing CVD risk as primary prevention, therefore those who did not have modifiable risk factors or who had pre-existing CVD, were excluded from the sample. Given that people with SMI are at an increased risk of CVD [2], it would be important for future studies to include those with a diagnosis of CVD as well as those who have yet to develop CVD risk factors to ensure that the results are generalisable to all people with SMI.

The primary outcome was attendance at primary care CVD risk reduction intervention appointments developed for the PRIMROSE research trial [33, 34]. While those who participate in research may not necessarily be representative of the population being studied; this study sample was drawn from 76 GP practices across diverse rural and urban settings in England. Also, attendance at research intervention appointments might not be the same as routine clinical care and research participants may be more motivated to attend. The intervention appointments were, however, delivered by practice nurses and HCAs working in GP practices providing CVD risk reduction advice and support in a clinical practice setting, rather than researchers employed on the study.

The finding that perceived social support was associated with attendance at PRIMROSE intervention appointments in the unadjusted but not adjusted analyses requires further exploration. The analysis may have been underpowered to detect a difference when multiple variables were added into the model, or there may be no true association between social support and appointment attendance once variables such as sex and age are taken into account.

The sample size for the study may not have been large enough to detect an association between social support and CVD risk-reducing health behaviours. The sample size only permitted inclusion of a small number of confounding variables, and multiple testing of secondary outcomes may have increased the risk of chance findings e.g., for adherence. The results for secondary outcomes should therefore be interpreted with caution and should be replicated in further research. There may have been additional confounding variables present that were not measured in the dataset. Variables such as level of education, socioeconomic status, severity of symptoms and negative symptoms are plausible factors that may influence perceived social support and participation in CVD risk-reducing behaviours.

Future work could assess the relationship between perceived social support and objective measures of health behaviours rather than self-report measures, and should aim to recruit a larger sample. Fewer inclusion criteria could be applied so that people with SMI with fewer CVD risk factors can be studied (e.g., those who are smokers or obese but who do not necessarily have raised cholesterol levels). Longitudinal analyses may also provide additional insights into the direction of associations.

To date, a number of recent studies in the field have tested the effectiveness of behavioural interventions on CVD health outcomes in SMI populations, few of which were superior to routine care on reducing CVD risk factors such as weight, smoking, HBA1c or cholesterol [35, 65, 66]. It may be that including a social support component would increase the uptake and adherence within these interventions, and further research is needed to clarify what form this should take, and to test its effectiveness. Further work is needed to identify effective intervention components that tackle the increasing health inequalities that people with SMI face in terms of their cardiovascular morbidity and mortality [2].

## Conclusion

Social support may be an important facilitator for adherence to CVD risk-reducing medications in people with SMI. Identifying people with low social support and exploring alternative ways to support them may help prevent disengagement with services and non-adherence to treatments; however, alternative strategies may be required to increase physical activity, improve diet and reduce smoking and alcohol intake in this population.

## Footnotes

1 The MMAS (8-item) content, name, and trademarks are protected by US copyright and trademark laws. Permission for use of the scale and its coding is required. A license agreement is available from Donald E Morisky, 14,725 NE 20th St Bellevue, WA 98,007, USA; dmorisky@gmail.com.
